# The specific DNA methylation landscape in focal cortical dysplasia ILAE type 3D

**DOI:** 10.1186/s40478-023-01618-6

**Published:** 2023-08-09

**Authors:** Dan-Dan Wang, Mitali Katoch, Samir Jabari, Ingmar Blumcke, David B. Blumenthal, De-Hong Lu, Roland Coras, Yu-Jiao Wang, Jie Shi, Wen-Jing Zhou, Katja Kobow, Yue-Shan Piao

**Affiliations:** 1 Department of Pathology, Xuanwu Hospital, Capital Medical University, No. 45, Changchun Street, Xicheng District, Beijing, 100053 China; 2 Clinical Research Center for Epilepsy, Capital Medical University, Beijing, 100053 China; 3 National Center for Neurological Disorders, Beijing, 100053 China; 4 Department of Neuropathology, Universitätsklinikum Erlangen, Friedrich-Alexander-Universität Erlangen-Nürnberg, Erlangen, Germany; 5 Biomedical Network Science Lab, Department of Artificial Intelligence in Biomedical Engineering, Friedrich-Alexander-Universität Erlangen-Nürnberg, Erlangen, Germany; 6 Department of Neurosurgery, Tsinghua University Yuquan Hospital, Beijing, 100049 China

**Keywords:** Epilepsy, Malformation of cortical development, Neuropathology, Occipital lobe, Epigenetic

## Abstract

Focal Cortical Dysplasia (FCD) is a frequent cause of drug-resistant focal epilepsy in children and young adults. The international FCD classifications of 2011 and 2022 have identified several clinico-pathological subtypes, either occurring isolated, i.e., FCD ILAE Type 1 or 2, or in association with a principal cortical lesion, i.e., FCD Type 3. Here, we addressed the DNA methylation signature of a previously described new subtype of FCD 3D occurring in the occipital lobe of very young children and microscopically defined by neuronal cell loss in cortical layer 4. We studied the DNA methylation profile using 850 K BeadChip arrays in a retrospective cohort of 104 patients with FCD 1 A, 2 A, 2B, 3D, TLE without FCD, and 16 postmortem specimens without neurological disorders as controls, operated in China or Germany. DNA was extracted from formalin-fixed paraffin-embedded tissue blocks with microscopically confirmed lesions, and DNA methylation profiles were bioinformatically analyzed with a recently developed deep learning algorithm. Our results revealed a distinct position of FCD 3D in the DNA methylation map of common FCD subtypes, also different from non-FCD epilepsy surgery controls or non-epileptic postmortem controls. Within the FCD 3D cohort, the DNA methylation signature separated three histopathology subtypes, i.e., glial scarring around porencephalic cysts, loss of layer 4, and Rasmussen encephalitis. Differential methylation in FCD 3D with loss of layer 4 mapped explicitly to biological pathways related to neurodegeneration, biogenesis of the extracellular matrix (ECM) components, axon guidance, and regulation of the actin cytoskeleton. Our data suggest that DNA methylation signatures in cortical malformations are not only of diagnostic value but also phenotypically relevant, providing the molecular underpinnings of structural and histopathological features associated with epilepsy. Further studies will be necessary to confirm these results and clarify their functional relevance and epileptogenic potential in these difficult-to-treat children.

**Supplementary Information:**

The online version contains supplementary material available at 10.1186/s40478-023-01618-6.

## Introduction

The International League Against Epilepsy (ILAE) proposed a clinico-pathological consensus classification system of Focal Cortical Dysplasia (FCD) in 2011 [6] and its revision in 2022 [36] to specify the histopathology landscape of FCD. FCD ILAE Type 3 was introduced to the classification scheme to address the many abnormalities in the neocortical architecture that were microscopically described in epilepsy surgery brain samples, including hippocampal sclerosis (FCD 3 A [47]), developmental brain tumors (FCD 3B [11, 14]), or vascular malformations (FCD 3 C [35, 51]). The fourth category of FCD 3D remained a ‘ragbag’ of architectural dysplasia associated with any other lesion acquired during early life and not assigned to FCD Type 3 A-C. These include mainly the various causes of pre- and perinatally acquired encephalomalacia and brain inflammation, i.e., Rasmussen encephalitis [50]. All FCD 3 subtypes share histopathology features of architectural dysplasia defined as abnormality in neocortical layering, representing either horizontal disorganization of layers 1 to 6 or representing vertical disorganization of the neocortex with excessive microcolumnar architecture or, most commonly, a mixture of both, horizontal and vertical cortical disorganization [5]. However, the underlying pathomechanism of a post-migratory acquired FCD remains enigmatic but was previously described as progressive cortical dysplasia due to postnatally delayed or otherwise compromised cortical maturation processes [33, 44].

An intriguing case cohort is that of perinatal hypoxic-ischemic injury occurring predominantly in boys with concomitant loss of layer 4 neurons in the occipital neocortex. This injury was recently classified as a distinct variant of FCD ILAE Type 3D [52]. However, this lesion pattern was initially described as a pathognomic example for FCD Type 1B in the ILAE classification scheme of 2011, i.e., the horizontal disorganization of the neocortex shown in Fig. 2C therein [6]. This example highlights the challenge of applying the FCD classification scheme without additional clinical information, as similar lesion patterns can define either FCD ILAE Type 1 or 3. The updated ILAE classification emphasizes that it is FCD Type 3 and its subtypes that recognize the abnormal architectural organization of the neocortex next to congenital epileptogenic lesions, such as developmental brain tumors, vascular malformations, or pre- and perinatal injuries [36]. In the mentioned case, the patient’s history of hypoxic-ischemic injury was initially not communicated to the neuropathologist, leading to a revised diagnosis much later only after integration of all available data [37].

**Fig. 1:**
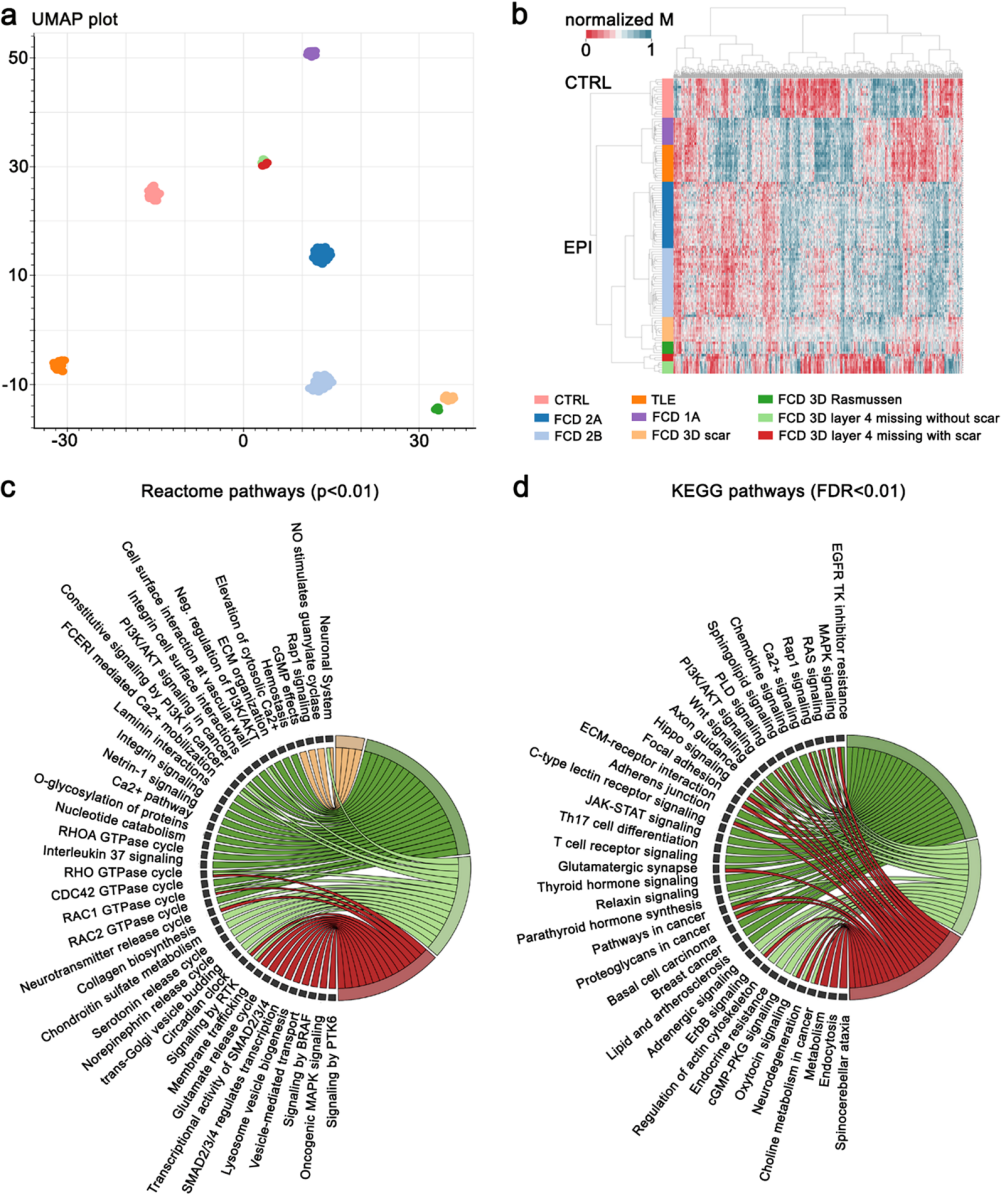
**a**:UMAP and **b**:hierarchical cluster analysis of FCD 3D subgroups together with FCD 2A/B, 1A, TLE, and autopsy controls. Histologically defined FCD 3D subgroups form separate and distinct methylation classes. **c**: Reactome and **d**: KEGG pathway enrichment of differentially methylated CpGs in FCD 3D subgroups. Each color represents a particular group, while a connection indicates whether a pathway is enriched. Pathways with two or more connections are enriched for two or more groups. The color scheme for histopathological entities in 1**a** also applies to 1**b**-**d**

While we have made considerable progress in understanding the molecular mechanisms of FCD ILAE Type 2, our current knowledge about clinical phenotypes and molecular signatures of patients with FCD ILAE Type 1 and 3 remains poor [4, 13]. Following the recently proposed DNA methylation-based CNS tumor classification applying commercially available 850 K/EPIC arrays for DNA extracted from archival formalin-fixed and paraffin-embedded tissue specimens [9], there is now also an ongoing interest in DNA methylation patterns in surgical epilepsy specimens to help with the pathologic diagnosis and correlate molecular findings with the clinical history [17, 19, 21].With its established role in gene regulation, DNA methylation has further received attention as disease mechanism contributing to the pathogenesis of epilepsy and associated structural brain lesions [15, 22, 23]. Here, we attempt to comprehensively describe the DNA methylation signature from surgical brain tissues, primarily focusing on FCD ILAE Type 3D with neuronal loss in layer 4.

## Materials and methods

### Ethics and study cohort selection

We included two patient cohorts in this study: One deriving from the department of Pathology, Xuanwu Hospital, Capital Medical University, and Yuquan Hospital, Tsinghua University in Beijing, China, and the other deriving from the Neuropathology Department at the University Hospital Erlangen in Germany.

*Beijing*: This study was reviewed and approved by the medical ethics committee of Xuanwu Hospital, Capital Medical University, before the study began (ethics board approval number: [2021]068). The surgical resection was decided after counseling the patient for treatment of their drug-resistant epilepsy and obtaining informed written consent from the patient or their legal guardians. The *Chinese cohort* included a retrospective series of 8 patients (7 males, 1 female) recruited between 2005 and 2019 who underwent electrocorticography (ECoG)-guided surgery for treatment of their drug-resistant epilepsy and were histopathologically diagnosed with FCD 3D associated with selective neuronal cell loss in layer 4 of the occipital lobe either without (n = 5; mean age ± SEM = 6.0 ± 1.1 years; Additional file 1: Fig. S1a-c) or with additional glial scars (*n* = 3; mean age ± SEM = 9.7 ± 1.5 years; Additional file 1: Fig. S1d-f) as described previously [52]. Perinatal adverse events, including early-life hypoxic injuries, were identified from medical histories in 8/8 patients.

*Erlangen*: Written informed consent for molecular-genetic investigations and publication of the results were obtained for all participating patients. The Ethics Committee of the Medical Faculty of the Friedrich-Alexander-University (FAU) Erlangen-Nürnberg, Germany, approved this study within the framework of the EU project “DESIRE” (FP7, grant agreement #602,531; ethics board approval numbers: AZ 92_14B, AZ 193_18B, and AZ 18–193_1-Bio). In the *German cohort*, we included previously published idat datasets from 112 individuals (58 males and 54 females) histopathologically diagnosed as FCD 1A (*n*= 11; mean age ± SEM = 9.9 ± 1.6 years), FCD 2A (*n*= 27; mean age ± SEM = 13.8 ± 1.98 years) and 2B (*n* = 28; mean age ± SEM = 18.43 ± 2.81 years), or temporal lobe epilepsy (TLE, *n* = 15; mean age ± SEM = 37.0 ± 3.95 years). All patients with TLE had a histopathological diagnosis of hippocampal sclerosis, and histopathologically normal temporal neocortex was used in the present analysis. Also, we included another FCD 3D cohort associated with glial scarring in encephalomalacia (*n* = 10; mean age ± SEM = 14.1 ± 3.16 years; Additional file 1: Fig. S1j-l) or early onset Rasmussen encephalitis (*n* = 5; mean age ± SEM = 18 ± 6.12 years; Additional file 1: Fig. S1g-i). Furthermore, we included non-epilepsy autopsy control cases with no known neurological history in the control cohort (*n* = 16; mean age ± SEM = 53.19 ± 4.7 years; previously unpublished control samples are summarized in Additional file 1: Table S1).

**Table 1:** Differentially methylated CpGs in FCD 3D and FCD 3D subtypes (adj. p-value < 0.05)

	Hypomethylated	Hypermethylated
FCD 3D all vs. Control	6154	1258
FCD 3D scar vs. Control	4883	2019
FCD 3D Rasmussen encephalitis vs. Control	46,107	12,743
FCD 3D with loss of layer 4 without scar vs. Control	42,087	9051
FCD 3D with loss of layer 4 and scar vs. Control	64,887	10,452

### Tissue preparation

For the Chinese cohort, resected brain tissues from cortical resections were immersion-fixed in 10% buffered formalin for histopathological examinations and cut perpendicularly to the cortical surface. Following routine paraffin embedding, 4 μm thin sections were stained with hematoxylin and eosin (H&E) and Luxol fast blue (LFB), as described previously [52]. H&E and immunohistochemical staining from all surgical specimens underwent systematic evaluation by two experienced neuropathologists. The blocks were selected according to H&E and immunohistochemical staining sections. According to the marking range of the sections on the blocks, the puncture was carried out on each block by a medullo-puncture needle (about 2 mm in diameter), with the fourth layer of the cortex as the center (including layer 4 with or without neuronal loss). The blocks were punctured 4–5 times in each case, and the tissue samples were taken for DNA extraction as specified below. For the German cohort, a prototypical area within the center of the lesion was microscopically identified on H&E slides, and tissue dissection was performed by punch biopsy (pfm medical, Köln, Germany) or manually from 10 adjacent unstained sections mounted on glass slides.

### Whole genome DNA methylation microarray

In the Chinese cohort, human DNA was extracted and purified from formalin-fixed paraffin-embedded (FFPE) tissues punches using QIAamp DNA FFPE Tissue Kit (QIAGEN, Cat No. Germany) according to the manufacturer’s instructions. The methylation of DNA was assayed on the Methylation 850K/EPIC Beadchip arrays (Illumina, San Diego, CA) using the Illumina HD methylation assay kit. It was performed at Shanghai Biotechnology Corporation following the manufacturer’s instructions. Total DNA (500 ng) was treated with bisulfate using an EZ DNA Methylation Gold Kit (Zymo Research, Irvine, CA).

In the German cohort, DNA was extracted from FFPE tissue using the Maxwell 16 FFPE Plus LEV DNA Kit (Promega, Madison, WI, USA), according to the manufacturer’s instructions. DNA concentration was quantified using the Qubit dsDNA BR Assay kit (Invitrogen, Carlsbad, CA, USA). Samples were analyzed at the Department of Neuropathology, Universitätsklinikum Heidelberg, Germany, using Illumina Infinium Methylation 850K/EPIC BeadChip arrays, as described previously [21]. Copy number profile analysis was assessed using the R package ‘conumee’ after an additional baseline correction (https://github.com/FAU-DLM/conumee).

### DNA methylation analysis

Differential DNA methylation analysis was performed with a self-customized Python wrapped cross R package pipeline as described [21]. Briefly, raw .idat methylation profiles were processed and subsequently normalized utilizing minfi’s Noob (normal-exponential out-of-band) normalization, a background correction method with dye-bias normalization for Illumina Infinium methylation arrays. Probes targeting sex chromosomes, probes containing single nucleotide polymorphisms (SNPs) not uniquely matching, as well as known cross-reactive probes (see [12]) were removed. We utilized our adapted ‘DNAmArray’ pipeline for probe filtering and imputation of missing values. Finally, 450,345 probes on the EPIC array were used for further analysis. As target variables, we identified the disease entities described above. These contained the FCD 3D cohort, TLEs, FCD 1A, 2A, and 2B, and no seizure autopsy controls. We histopathologically distinguished four groups within the FCD 3D cohort as described above. Most significantly differentially methylated CpGs between these entities were identified by fitting a regression model with these groups as the target variable using the ‘limma’ R package. All pairwise comparisons between these groups were identified as contrasts and included in the analysis. We identified three surrogate variables, which we adjusted for (‘sva’ R package). In addition, we included the age of onset and duration of epilepsy in our model design. Finally, we corrected for batch number, neuronal proportion, the brain region the specimen originated from, and center (removeBatchEffect: ‘limma’ R package.). After identifying 683 unique, most significantly differentially methylated CpGs (adj. *p*-value < 0.05, absolute logFC > 2), unsupervised dimensionality reduction for cluster analysis was performed. Uniform Manifold Approximation and Projection (UMAP) for general non-linear dimensionality reduction was used for visualization [26]. After identifying disease clusters, care was taken that no cluster was confounded or correlated with any other variable such as sex, age at onset, age at surgery, duration of epilepsy, or lobe (Additional file 1: Fig. S2). Additional hierarchical cluster analysis was performed.

### Functional pathway enrichment analysis

Significantly differentially methylated CpGs were used for pathway enrichment analyses to identify pathways more likely to be enriched for differential methylation. Our enrichment analysis was implemented with the missMethyl R package via the ‘GOmeth’ function, using gene ontology (GO) terms from the GO.db annotation package and Kyoto Encyclopedia of Genes and Genomes (KEGG) pathways from the KEGG.db annotation package [32, 40]. The above-identified 450,345 probes obtained after preprocessing and normalization were tested as background/universe. Additionally, we performed REACTOME gene sets enrichment using the ‘gsameth’ function which takes the user-supplied list of gene sets to be tested for enrichment. The REACTOME gene set was retrieved from the msigdbr R package [28]. missMethyl takes care of biases such as the heterogenous distribution of probes per gene present on the array, and multiple genes annotated to one CpG while performing the enrichment for significant CpGs from the methylation array [31]. KEGG pathways with FDR < 0.05 and REACTOME pathways with *p* < 0.01 were kept. We used GOplot to display the relationship between entities and KEGG or REACTOME terms [49].

### Differentially methylated genes and de novo network enrichment

We used the mCSEA R package to identify differentially methylated regions. It was reported to be especially useful for detecting subtle but consistent methylation differences in complex phenotypes [34]. Starting from our matrix of β-values the rankProbes() functiolonging to the same region in the top positions of the ranked list. Regions whose CpG sites were over-represented in the top or bottom of the list were detected as differentially methylated and mapped to gene promoters (1,500 bp upstream TSS). We then used the web interface (https://robust-web.net) of the ROBUST disease module mining tool [2] to uncover candidate disease mechanisms for the FCD 3D subgroups. For this, we downloaded a human annotated protein-protein interaction (PPI) network from the Integrated Interactions Database [24] and then filtered the network to keep only experimentally confirmed PPIs where both interaction partners are expressed in brain tissue. The resulting brain-specific PPI network was then used as input for the ROBUST tool, together with the list genes with differentially methylated promoter regions in the FCD 3D with loss of layer 4 cohort. ROBUST returned a module with 182 nodes. 147 nodes fall into the largest connected component (LCC) and were further used for our analyses; the remaining unconnected nodes were discarded. We used DIGEST [1] to *in silico* validate the obtained LCC w.r.t. functional coherence. Given a set of input genes, DIGEST computes empirical *p*-values, comparing the distributions of pairwise concordances of the genes’ GO and KEGG annotations against a random background model. Moreover, we carried out gene set enrichment analysis on the module’s LCC via g:Profiler [41] and ranked the genes within the LCC via their PageRank centralities [8], which we computed using the implementation provided in NetworkX [45].

### Data and code availability

Methylation data were deposited at GEO for the German cohort (https://www.ncbi.nlm.nih.gov/geo/, under the accession numbers GSE185090, GSE156374, and GSE227239) or at OMIX for the Chinese cohort (https://ngdc.cncb.ac.cn/omix, under the accession number PRJCA009245). Additional file 1: Table S1 summarizes the idats for FCD3D and previously unpublished control cases. Code and instructions to reproduce the *de novo*network enrichment analysis are available on GitHub: https://github.com/bionetslab/fcd3-methylation-landscape.

## Results

### DNA methylation defines FCD 3D in unprecedented granularity

To determine whether DNA methylation signatures can be used to classify structurally highly related lesions at the molecular level, we used DNA methylation data from surgical brain samples obtained from 104 patients with drug-resistant epilepsy and a histopathological diagnosis of FCD ILAE Type 3D (*n* = 23), FCD ILAE Type 1A (*n* = 11), FCD ILAE Type 2A (*n* = 27), FCD ILAE Type 2B (*n* = 28), and TLE (*n* = 15) compared to 16 autopsy controls without any seizures in their clinical history (CTRL). A total of 6,154 hypomethylated and 1,258 hypermethylated probes with adjusted *p*-value < 0.05 were identified between FCD 3D and control (Table 1). As previously described, we performed unsupervised dimensionality reduction and hierarchical cluster analysis and identified FCD and TLE-specific methylation classes in the UMAP dimensionality reduction [23]. Our data further provided evidence for high granularity in histopathology-specific DNA methylation signatures. UMAP clustering identified three separate methylation classes within the FCD 3D family of samples, matching with loss of layer 4 (*n* = 8), Rasmussen encephalitis (*n*= 5), and scar (*n*= 10; Fig. 1a). No confounding correlation with any other variable of our data was detected (e.g., sex, age, lobe; Additional file 1: Fig. S2). Hierarchical cluster analysis confirmed the separation of samples at the disease level, including a further distinction between FCD 3D with loss of layer 4 with or without adjacent scars (Fig. 1b, Additional file 1: Fig. S1). Taken together, our data indicate that DNA methylation signatures define FCD 3D and its histopathological subgroups in unprecedented granularity.

### Functional pathway analysis identifies specific disease mechanisms in FCD 3D

Next, we performed functional pathway analysis. We mapped differentially methylated CpGs and the respective genes to KEGG (FDR < 0.01) and REACTOME pathways (*p*  < 0.01). We found that differential DNA methylation in FCD 3D targeted functional pathways in a histopathological subgroup-specific manner. In FCD 3D with loss of layer 4, differential DNA methylation uniquely targeted functional pathways related to *Neurodegeneration*, *MAP Kinase signaling* (*BRAF signaling, RTK signaling*), *Collagen biosynthesis*,*Chrondroitin sulfate metabolism*,and *Regulation of actin cytoskeleton* (Fig. 1c-d). In contrast, FCD 3D with Rasmussen encephalitis yielded differential DNA methylation enrichment in *Ca2 + signaling, PI3K/AKT signaling, ECM organization*, *ECM receptor interactions* (*Integrin signaling, C-type lectin receptor signaling*), *Netrin signaling*, and *Laminin interactions.* Pathways jointly affected in Rasmussen encephalitis and loss of layer 4 associated FCD 3D included *Axon guidance*, *Focal adhesion*, *Adherence junction*, and *Hippo signaling* (Fig. 1c-d). Intriguingly, this particular approach yielded no pathway-specific enrichment in FCD Type 3D associated with scars despite almost 7,000 differentially methylated CpGs (Table 1; Fig. 1c-d). Our data suggest that differential DNA methylation in FCD 3D targeted distinct genes and pathways in FCD 3D subgroups.

### Differential promoter methylation further highlights specific disease targets

**Fig. 2:**
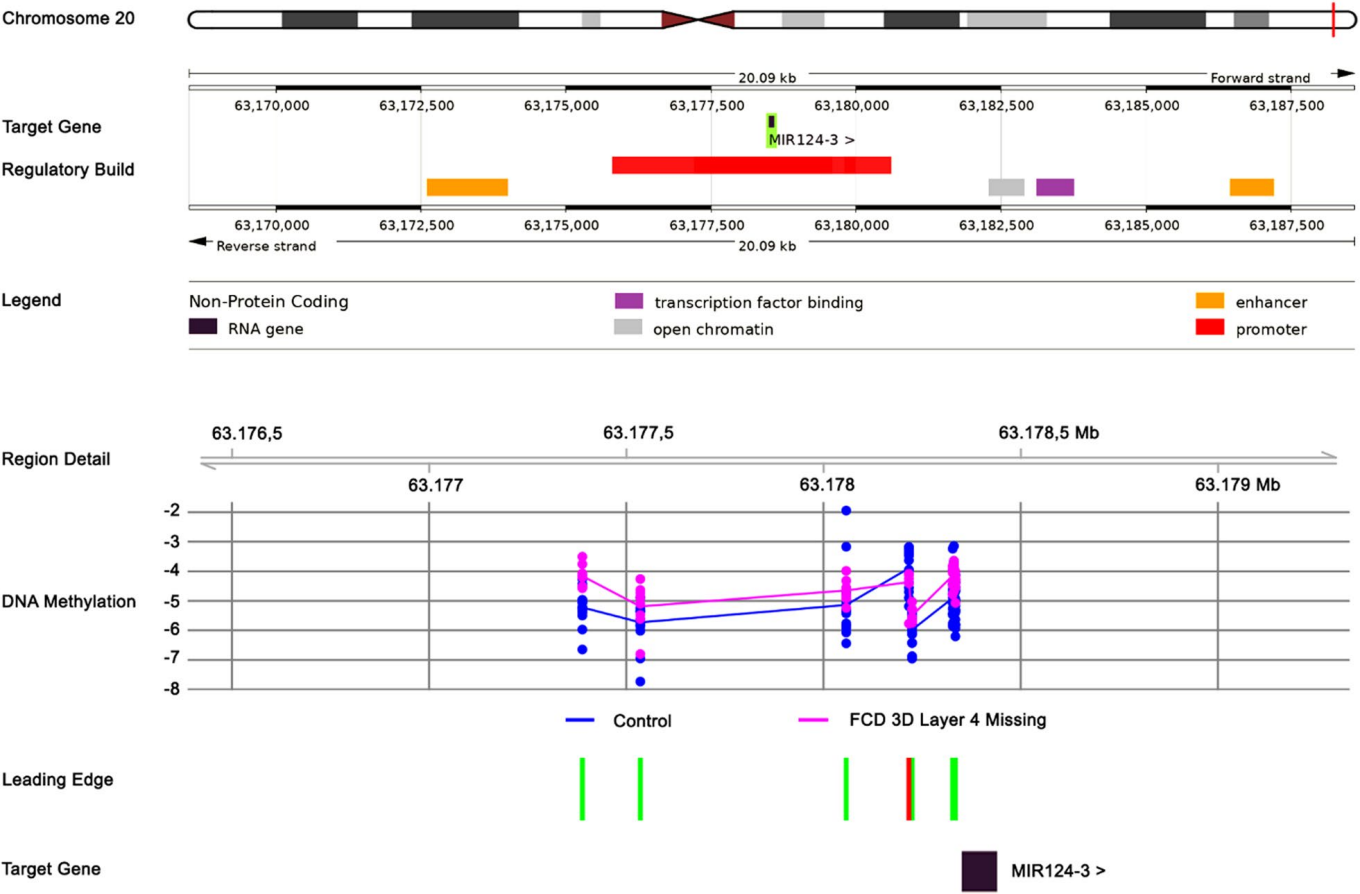
Differential promoter methylation of mir-124-3 in FCD 3D with loss of layer 4. The figure summarizes from top to bottom the genomic environment of the mir-124-3 gene with strand specificity on chromosome 20, gene regulatory features based on ENSEMBL annotations, including putative promoter and enhancer regions, and increased promoter methylation in FCD 3D with loss of layer 4. Leading edge indicates CpGs with significant and uniform methylation change (here increase) in green upstream of the transcriptional start site of miR-124-3. CpGs not considered are marked in red

To further explore the functional relevance of DNA methylation changes in FCD 3D subgroups, we mapped differentially methylated regions with ≥ 5 adjacent differentially methylated CpGs to gene promoters using the mCSA tool [34]. In FCD 3D with loss of layer 4 with or without scars, DMRs mapped to 182 gene promoters (FDR < 0.01), including RNA (e.g., has-mir-124-3, has-mir-886; Figs. 2) and 177 protein-coding genes. In FCD 3D with Rasmussen encephalitis, only 66, and in FCD 3D with scars, 132 gene promoters were found to be differentially methylated. There was minimal overlap between differentially methylated genes between FCD 3D subgroups with only one gene shared between all three subgroups (i.e., *Homeobox A3*, HOXA3).

**Fig. 3:**
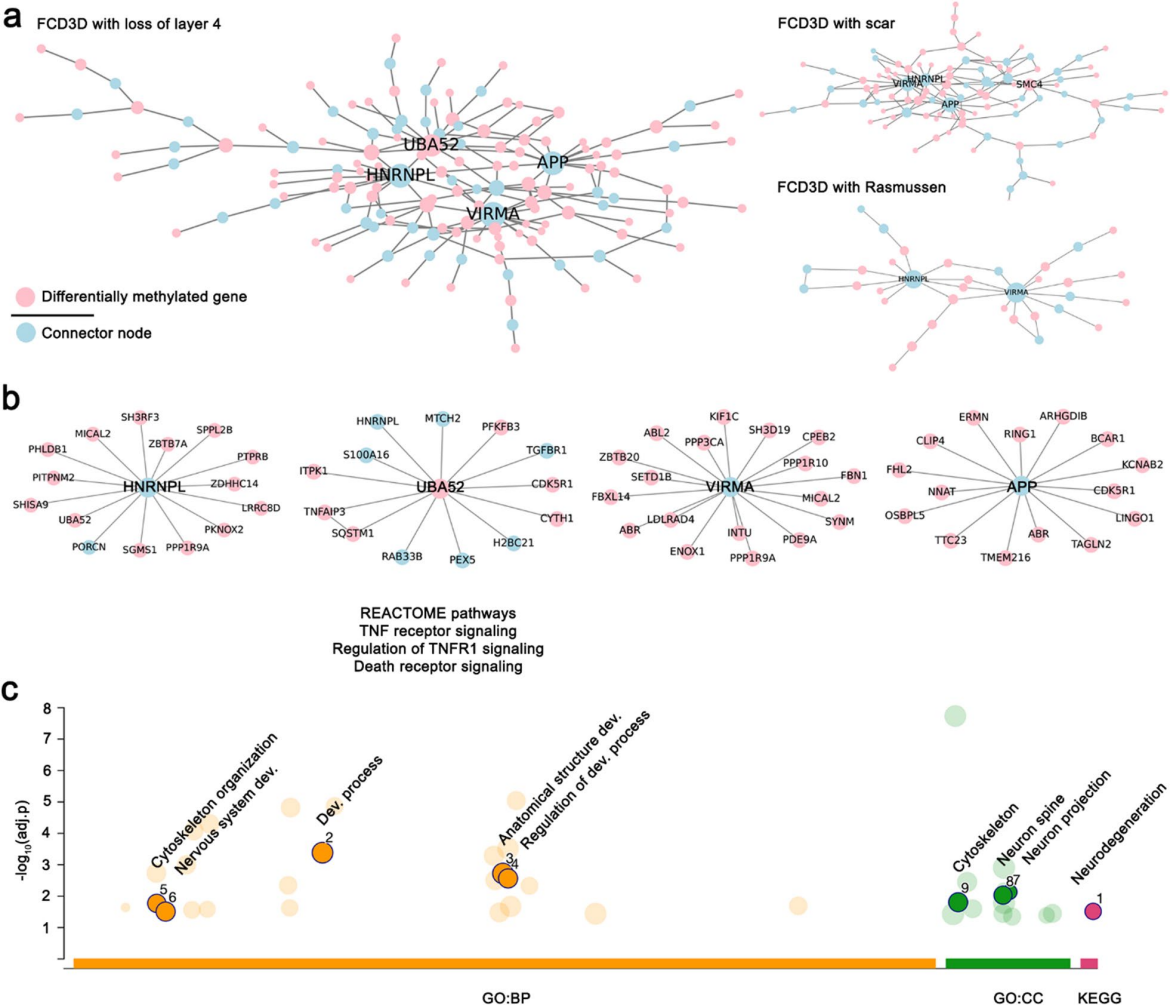
Protein-protein interaction (PPI) and functional enrichment of differentially methylated genes in FCD 3D with loss of layer 4. 3**a** PPI networks of FCD 3D subgroups. Differentially methylated genes are marked in pink, and connector nodes are shown in blue. The loss of layer 4 subgroup showed the largest connected PPI network with four major topological modules. **b** They centered around HNRNPL, VIRMA, UBA52, and APP. The UBA52 module significantly overrepresented proteins involved in TNF and death receptor signaling. **c** Functional enrichment of the FCD 3D with loss of layer 4 PPI network highlighted neurodegeneration (KEGG), cytoskeleton organization, and nervous system and anatomical structure development (GO:BP) mainly targeting neuron projections and spine (GO:CC) as central disease mechanisms. APP - Amyloid Beta Precursor Protein, BP – Biological Process, CC – Cellular Component, Dev. – development/developmental, FCD – Focal Cortical Dysplasia, GO – Gene Ontology, HNRNPL -Heterogeneous Nuclear Ribonucleoprotein L, KEGG – Kyoto Encyclopedia of Genes and Genomes, TNF – Tumor Necrosis Factor, UBA52 - Ubiquitin A-52 Residue Ribosomal Protein Fusion, VIRMA - Vir Like M6A Methyltransferase Associated

We next analyzed protein-protein interactions (PPI) in the differentially methylated histopathology-specific target gene sets to further uncover candidate disease mechanisms. Thereby, we concentrated only on experimentally confirmed and not predicted PPIs where both interaction partners are reported to be expressed in brain tissue [24]. Then we used the disease module mining tool ROBUST [2]. Hyper-parameters were adjusted to require high confidence for the algorithm to include a connector node in the results and correct for study bias in PPI networks. The PPI network derived from FCD 3D with loss of layer 4 consisted of 182 nodes, including 129 differentially methylated genes (seeds, pink) and 53 connector nodes (targets, blue), and 185 edges. One hundred forty-seven nodes fell into the largest connected component (LCC) and were further used for analyses (Fig. 3a, left panel). The remaining unconnected nodes were discarded. The functional coherence of this PPI network was higher than in random reference networks of similar size (Additional file 1: Fig. S3). Also, it differed from that of other FCD 3D subgroups (Fig. 3a, right panel).

Specific topological modules, based on nodes with significantly more connections centered around *Heterogeneous Nuclear Ribonucleoprotein L* (HNRNPL), a protein involved in the formation, packaging, processing, and function of mRNA; *Ubiquitin A-52 Residue Ribosomal Protein Fusion* (UBA52), a protein involved in proteasomal degradation; *Vir Like M6A Methyltransferase Associated* (VIRMA), a protein involved in mRNA alternative polyadenylation and mRNA methylation; and *Amyloid Beta Precursor Protein* (APP), a neuronal cell surface receptor relevant to neurite growth, neuronal adhesion, and axonogenesis as well as synaptogenesis, cell mobility, and transcription regulation (Fig. 3c). Thereby, HNRNPL, VIRMA, and APP were connector nodes without disease-related changes in DNA methylation of their own, but linking large numbers of differentially methylated genes. Looking further into the functional role of topological modules using g:Profiler, we found an overrepresentation of proteins from the UBA52 module in the REACTOME pathways *PMID: 28,942,967*, *Regulation of TNFR1 signaling*, and *Death receptor signaling*, thus, linking this module to TNFR1-mediated apoptosis and inflammation. The HNRNPL, VIRMA, and APP modules had no overrepresentation of specific pathways. Functional enrichment analysis from the entire largest connected component identified the KEGG pathway *Neurodegeneration in multiple diseases* as constituting candidate disease mechanisms targeted by DNA methylation changes in FCD 3D with loss of layer 4 (Fig. 3c). Key differentially methylated genes implicated in neurodegeneration included Wnt family member 6 (WNT6), Frizzled Class Receptor 7 (FZD7), Calmodulin-like 3 (CALML3), Caspase 8 (CASP8), Cyclin-dependent kinase 5 regulatory subunit 1 (CDK5R1), Cytochrome c oxidase subunit 7A1 (COX7A1), Protein phosphatase 3 catalytic subunit alpha (PPP3CA also known as calcineurin), Sequestosome 1 (SQSTM1), and UBA52.

## Discussion

Our understanding of the molecular basis of FCD has changed dramatically during the past ten years, particularly for FCD ILAE Type 2, with ample scientific evidence for pathogenic and epileptogenic brain somatic mutations in genes of the mTOR pathway [3, 25, 29]. Likewise, mild malformations of cortical development with oligodendroglial hyperplasia have been proven to be SLC35A2 altered [5, 7]. In contrast, the genetic background of FCD Type 1 and 3 remains elusive [18]. DNA methylation analysis may be used as an alternative strategy for the molecular classification of histopathological entities and help understand underlying disease mechanisms when pathogenically relevant genomic variants cannot be identified [19, 21]. The present study establishes DNA methylation analysis as a suitable tool to molecularly separate microscopically different subtypes of FCD Type 3, either associated with selective loss of layer 4, glial scarring in encephalomalacia, or Rasmussen encephalitis. Further mapping of differential DNA methylation to functional pathways helped gain initial insights into the biological meaning of the observed methylation changes in alignment with structural and functional changes observed in FCD Type 3D.

Epigenetic signatures are emerging as diagnostic biomarkers for focal structural epilepsy [3, 18, 19, 21]. Methylation sequencing in FCD revealed differential methylation in intergenic regions, gene bodies, and enhancers, distinguishing it from promoter-centered DNA methylation changes in brain tumors [23]. DNA methylation-based hierarchical cluster analysis can differentiate various cortical malformations and may contribute to an integrated diagnostic classification scheme for epilepsy-associated MCD [19]. Further work also established DNA methylation-based detection of chromosomal copy number variation as a practical application to molecularly and clinically stratify patients with brain malformations [21]. The present study molecularly differentiated the family of FCD ILAE Type 3D lesions in unprecedented detail, and provided some mechanistic insights for this debated entity [52].

Histopathologic FCD diagnosis can be difficult, and the classification scheme has been modified over time to account for that [6, 37, 39, 46]. In 2011, ILAE released the first international consensus classification of FCD in order to create a pathology-based subdivision of FCD with distinct clinical representations and outcomes [6]. However, challenges remained regarding the diagnoses of FCD 1 and 3 [5]. The most recent ILAE classification update included an integrated genotype-phenotype scheme [36], but there is a considerable knowledge gap for FCD 1 and 3 subtypes without gene-driving mutations. Our data support the notion that DNA methylation–based classification may become helpful in routine diagnostics as it reliably distinguishes FCD subtypes and provides some insights into their underlying biology.

In the present study, we identified three subtypes of FCD 3D, histopathologically characterized by (i) glial scarring, (ii) selective loss of layer 4, or (iii) Rasmussen encephalitis, all with a distinct DNA methylation signature. Functional pathway enrichment analysis consistently showed that in FCD 3D characterized by neuronal cell loss in cortical layer 4, differential DNA methylation targeted *neurodegeneration pathways*, *apoptosis*, and *TNF* and *death receptor signaling*. These data suggest that the observed loss of layer 4 is cell death associated and not the consequence of a migration defect and misplacement of cells.

TNF receptor signaling is also linked to inflammation and epilepsy. Pathological events initiated in the CNS by local injuries (e.g., hypoxic-ischemic injury, trauma, stroke) or peripherally following infections or autoimmune-mediated can lead to activation of neurons, glia, or leukocytes, respectively. These cells release inflammatory mediators into the brain or blood, eliciting a cascade of inflammatory events that cause a spectrum of physiopathological outcomes. Evidence from rodent models suggest an active role for TNF and other inflammatory factors in seizure generation [48]. In our study, only the FCD 3D subgroup with loss of layer 4 showed functional enrichment in TNF receptor signaling. However, the study of surgical brain tissue provides only a molecular and structural glimpse into a late and chronic disease stage. Also, we only investigated DNA methylation and no gene expression or other molecular layers of information. It cannot be ruled out, therefore, that other FCD 3D subgroups may share altered TNF receptor signaling at different disease stages.

We further found unique promoter methylation changes in FCD 3D with loss of layer 4, which targeted non-coding RNAs, including has-mir-124-3. MicroRNA miR-124-3 has been reported to play a role in the development of epilepsy. Studies have shown that it regulates genes involved in synapse formation and synaptic plasticity. The latter is the ability of synapses to change strength in response to changes in neural activity. This suggests that miR-124-3 may regulate the excitability of neurons and, subsequently, the formation of seizures.

DNA methylation changes also targeted pathways involved in *Axon guidance*, *Regulation of the actin cytoskeleton, Focal adhesion*, and *Adherens junction*. Axon guidance is a key process in forming neuronal networks during CNS development [38]. It can be reactivated after early-life injury to repair lesioned circuits [42]. However, misguided axons are likely to contribute to epileptic network formation [30]. Axon growth and guidance depend on the actin cytoskeleton as well as the composition and viscoelastic properties of the surrounding ECM. Disruption in the actin cytoskeleton in the brain gives rise to malformations of cortical development and epilepsy [27]. The brain ECM also plays a critical role in governing brain structure, excitability, and function. Intriguingly, specific pathways related to *Proteoglycans*, *Chondroitin sulfate metabolism*, *O-glycosylation of proteins*, and *Laminin signaling* were targeted by differential DNA methylation indicating broad *ECM organization* changes in FCD 3D. Finally, DNA methylation alterations impacted *Integrin* and *Hippo signaling*, both involved in mechanosensing and cell adhesion [10, 43], as well as *Adherens junctions*, critical for cell-cell or cell-matrix contacts and essential components of the blood-brain barrier [20].

In conclusion, this is the first study integrating DNA methylation to classify FCD 3D, focusing on the subtype previously described by selective neuronal cell loss in layer 4. Our study supported the need for an in-depth comparative analysis of related FCD subtype families as they may represent a diverse spectrum of etiologies and molecular-genetic backgrounds. The expression of axon guidance molecules and their receptors, as well as the physicochemical role of the ECM, may become a new topic of interest for this FCD entity but requires further work, including targeted animal models [16, 30, 42]. Furthermore, it remains to be shown, if a common and open access FCD DNA methylation classifier will be made available in the near future to support the diagnostic yield of difficult-to-classify FCD, such as FCD ILAE 1 and 3 subtypes.

### Supplementary Information

**Additional file 1:** Supplementary figures and tables.
