# Correction to: The specific DNA methylation landscape in focal cortical dysplasia ILAE type 3D

**DOI:** 10.1186/s40478-024-01752-9

**Published:** 2024-03-29

**Authors:** Dan‑Dan Wang, Mitali Katoch, Samir Jabari, Ingmar Blumcke, David B. Blumenthal, De‑Hong Lu, Roland Coras, Yu‑Jiao Wang, Jie Shi, Wen‑Jing Zhou, Katja Kobow, Yue‑Shan Piao

**Affiliations:** 1 Department of Pathology, Xuanwu Hospital, Capital Medical University, No. 45, Changchun Street, Xicheng District, 100053 Beijing, China; 2 Clinical Research Center for Epilepsy, Capital Medical University, 100053 Beijing, China; 3 National Center for Neurological Disorders, 100053 Beijing, China; 4 Department of Neuropathology, Universitätsklinikum Erlangen, Friedrich- Alexander-Universität Erlangen-NÜrnberg, Erlangen, Germany; 5 Biomedical Network Science Lab, Department of Artificial Intelligence in Biomedical Engineering, Friedrich-Alexander-Universität Erlangen-NÜrnberg, Erlangen, Germany; 6 Department of Neurosurgery, Tsinghua University Yuquan Hospital, 100049 Beijing, China

**Correction to:** ***acta neuropathol commun*** **(2023) 11:129**

10.1186/s40478-023-01618-6

Following publication of the original article [1], the authors identified errors in the reference list and citations. Due to a technical error a wrong article version for the references and citations was used.

The original article [1] has been corrected.
