# Supplementary material for: The specific DNA methylation landscape in focal cortical dysplasia ILAE type 3D
Source: Acta Neuropathol Commun. 2023 Aug 9;11:129. doi: 10.1186/s40478-023-01618-6 (PMC10410964; doi:10.1186/s40478-023-01618-6)
Supplement: Supplementary file 1 — Additional file 1: Supplementary figures and tables. [file 40478_2023_1618_MOESM1_ESM.docx]

**Supplementary Material**

**Supplement Figure 1**


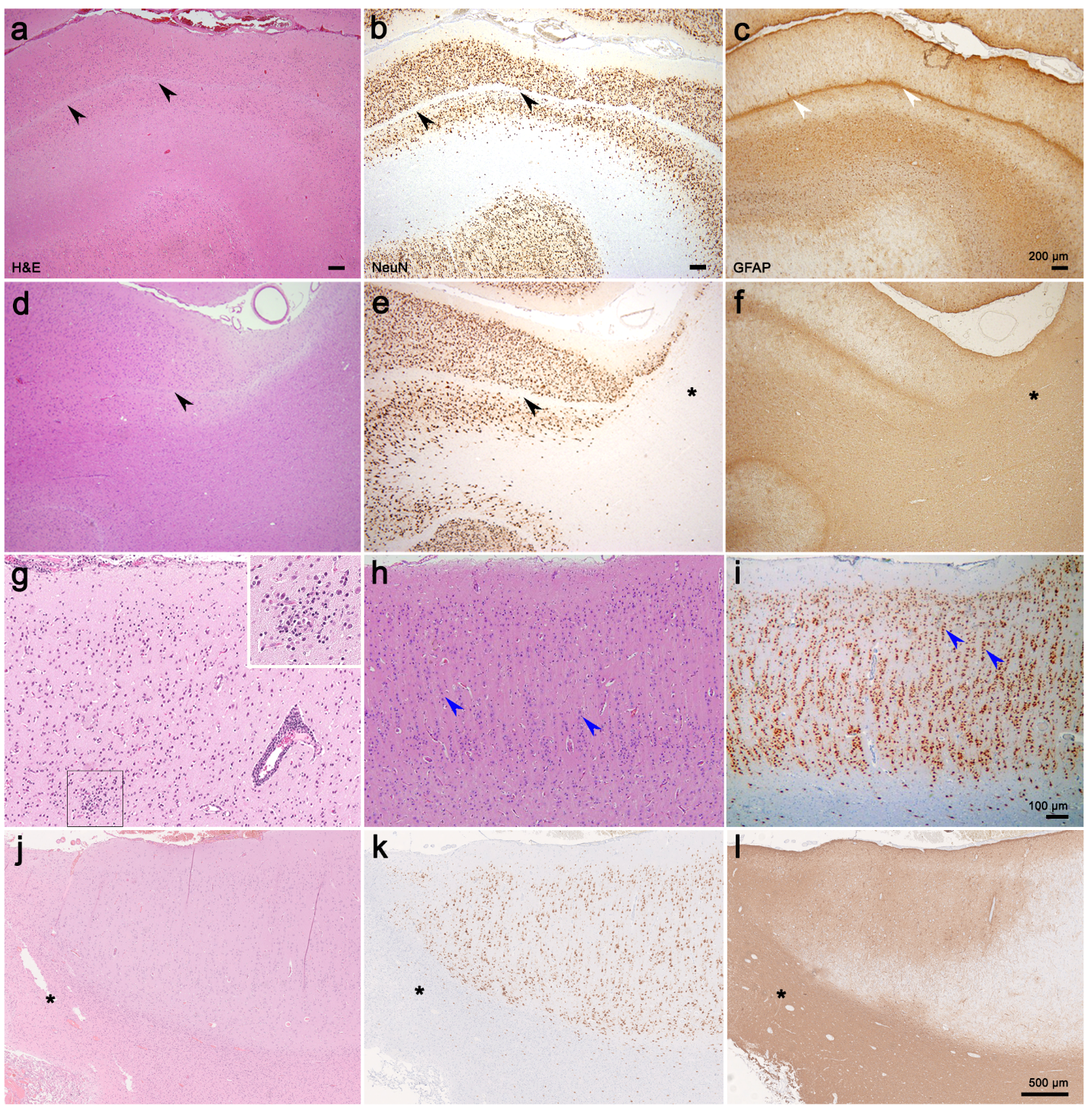


**Legend to Supplement Figure 1**: **Histopathology of FCD Type 3D subgroups**. (**a-c**) Recently described FCD 3D subgroup of samples characterized by selective neuronal loss in layer 4 (black arrowheads), (**d-f**) sometimes accompanied by a scar (asterisk). (**g-i**) In contrast, histopathological patterns in Rasmussen encephalitisare characterized by multifocal distribution of perivascular lymphocyte cuffing and discrete microglial nodule formation (insert).FCD 3D accompanying Rasmussen encephalitis is marked by abnormal radial architecture in the cortex (blue arrowheads) without inflammatory change. (**j-l**) FCD 3D with extensive scarring (asterisk) as seen, e.g., following traumatic brain injury, intrauterine infarctions, or repeat surgery.

**Supplement Figure 2**

**
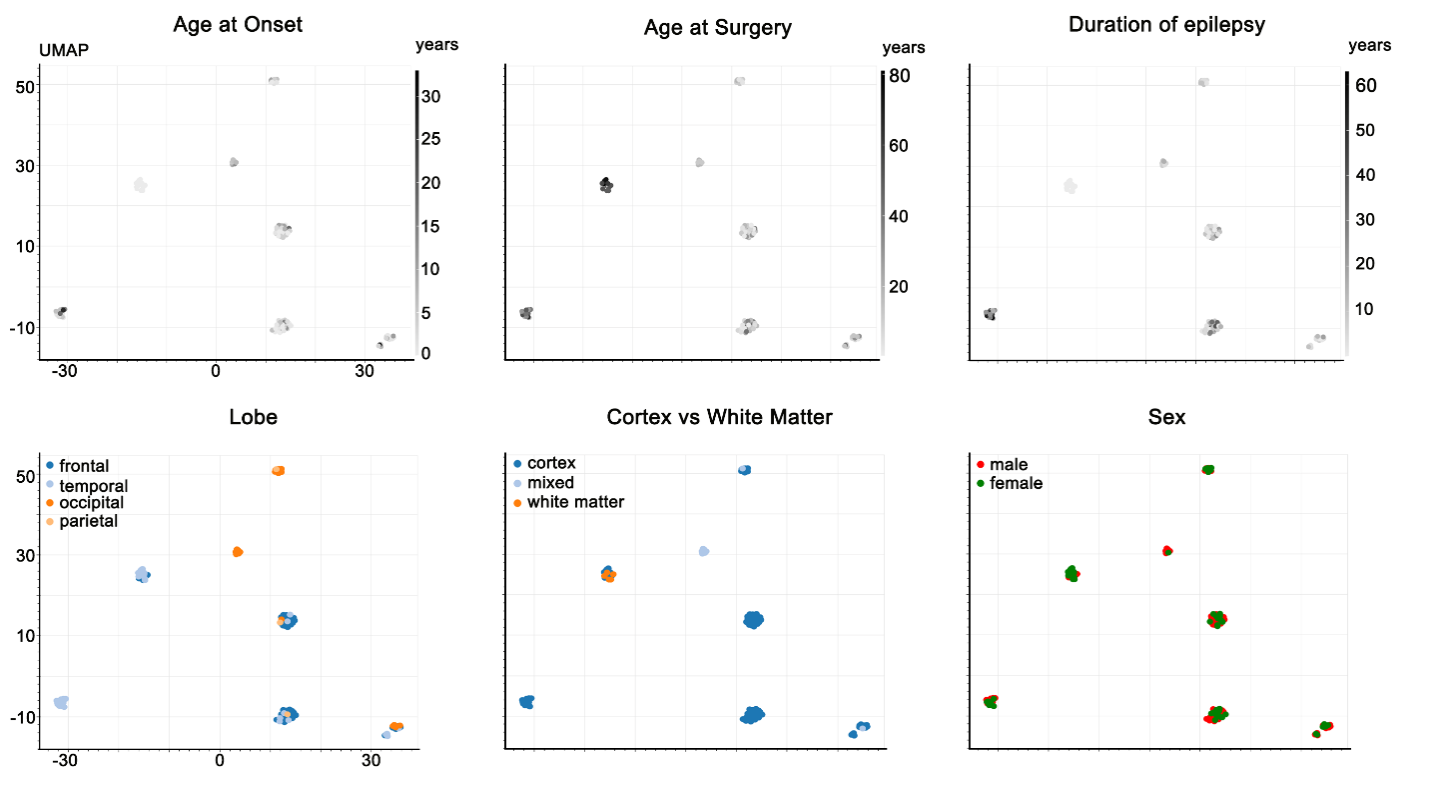
Legend to Supplement Figure 2**: UMAP plots from most significantly differentially methylated positions (as in Fig. 1a) overlayed with potentially confounding variable influence. Samples were labeled with the covariates age at onset, age at surgery, duration of epilepsy, sampled lobe, cortex or white matter, and sex. The tested covariates did not drive the clustering of samples.

**Supplement Figure 3**


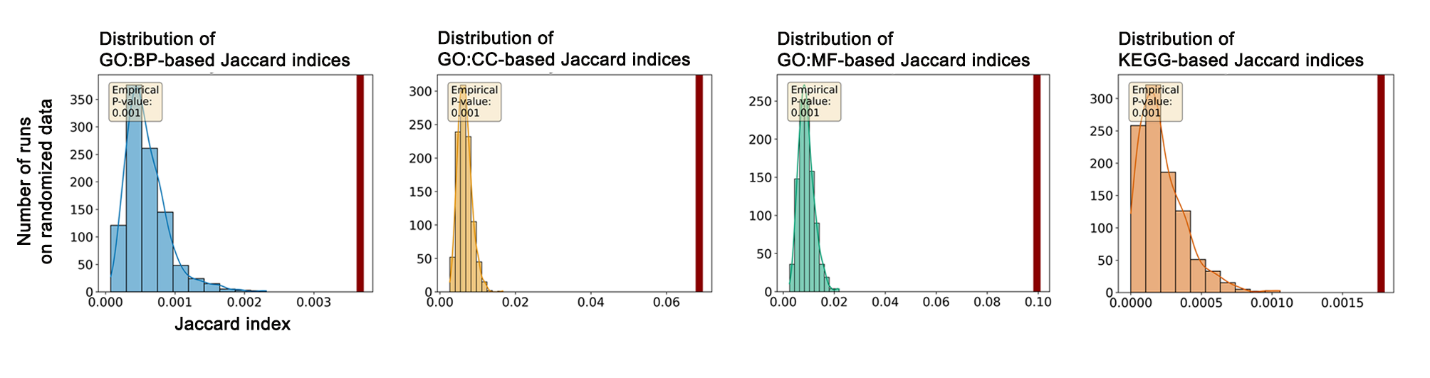


**Legend to Supplement Figure 3**: Comparison of the functional coherence of the genes contained in the largest connected component of the PPI module computed by ROBUST run with the set of FCD 3D with loss of layer 4 differentially methylated genes as input against a random background model. Functional coherence is quantified as the mean pairwise Jaccard index of the genes. For all functional annotation classes (i.e., GO:BP, GO:CC, GO:MF, and KEGG pathways), higher mean Jaccard indices were obtained for the PPI module derived from the differentially methylated genes in FCD 3D with loss of layer 4 (bold red lines) than for random reference gene sets with matched numbers of functional annotations (bars and thin fitted curves). BP – Biological Process, CC – Cellular Component, FCD – Focal Cortical Dysplasia, GO – Gene Ontology, KEGG – Kyoto Encyclopedia of Genes and Genomes, MF – Molecular Function, PPI – Protein-Protein-Interaction.

**Supplement Table 1:** Clinical summary of FCD3D cohort and unpublished controls.

| **pathology** | **lobe** | **sex** | **age_onset** | **age_surgery** | **duration** | **idat** |
| --- | --- | --- | --- | --- | --- | --- |
| Control - NCx | temporal | M | 0.0 | 68 | 0.0 | 205982890094_R05C01 |
| Control - NCx | temporal | F | 0.0 | 81 | 0.0 | 205982890094_R06C01 |
| Control - NCx | temporal | F | 0.0 | 57 | 0.0 | 205982890094_R07C01 |
| Control - NCx | temporal | F | 0.0 | 72 | 0.0 | 205982890094_R08C01 |
| Control - NCx | temporal | F | 0.0 | 75 | 0.0 | 205982890108_R01C01 |
| Control - NCx | temporal | F | 0.0 | 61 | 0.0 | 205982890108_R02C01 |
| Control - NCx | temporal | F | 0.0 | 56 | 0.0 | 205982890108_R03C01 |
| FCD 3D with loss of layer 4 | occipital | M | 5.0 | 12.0 | 7.0 | 203712390068_R06C01 |
| FCD 3D with loss of layer 4 | occipital | M | 6.0 | 12.0 | 6.0 | 203789410114_R04C01 |
| FCD 3D with loss of layer 4 | occipital | M | 6.0 | 18.0 | 12.0 | 203712390068_R04C01 |
| FCD 3D with loss of layer 4 | occipital | M | 9.0 | 17.0 | 8.0 | 203712390068_R08C01 |
| FCD 3D with loss of layer 4 | occipital | M | 4.0 | 20.0 | 16.0 | 203789410114_R02C01 |
| FCD 3D with loss of layer 4 | occipital | F | 13.0 | 17.0 | 4.0 | 203789410114_R07C01 |
| FCD 3D with loss of layer 4 | occipital | M | 9.0 | 17.0 | 8.0 | 203789410114_R08C01 |
| FCD 3D with loss of layer 4 | occipital | M | 7.0 | 12.0 | 5.0 | 203789410114_R06C01 |
| FCD 3D-scar | frontal | F | 10.0 | 17.0 | 7.0 | 203219730159_R01C01 |
| FCD 3D-scar | frontal | M | 0.5 | 5.0 | 4.5 | 203219730159_R02C01 |
| FCD 3D-scar | temporal | M | 1.0 | 2.0 | 1.0 | 203219730159_R03C01 |
| FCD 3D-scar | occipital | M | 11.0 | 31.0 | 20.0 | 203219730159_R06C01 |
| FCD 3D-scar | occipital | F | 0.0 | 31.0 | 0.0 | 203219730159_R05C01 |
| FCD 3D-scar | frontal | F | 0.0 | 14.0 | 0.0 | 203219730159_R07C01 |
| FCD 3D-scar | frontal | F | 3.3 | 7.0 | 3.7 | 203219730055_R08C01 |
| FCD 3D-scar | occipital | M | 3.0 | 12.0 | 9.0 | 203219730159_R04C01 |
| FCD 3D-scar | occipital | F | 0.6 | 14.0 | 13.4 | 203219730159_R08C01 |
| FCD 3D-scar | frontal | M | 1.0 | 8.0 | 7.0 | 203219640118_R06C01 |
| FCD 3D  Rasmussen | temporal | M | 7.0 | 11.0 | 4.0 | 203219640118_R07C01 |
| FCD 3D  Rasmussen | temporal | F | 33.0 | 42.0 | 9.0 | 203219640218_R03C01 |
| FCD 3D  Rasmussen | frontal | M | 3.3 | 14.0 | 10.7 | 203219640118_R08C01 |
| FCD 3D  Rasmussen | frontal | F | 6.0 | 15.0 | 9.0 | 203219640218_R01C01 |
| FCD 3D  Rasmussen | temporal | M | 5.0 | 8.0 | 3.0 | 203219640218_R02C01 |
